# Enhanced three-photon activity triggered by the AIE behaviour of a novel terpyridine-based Zn(ii) complex bearing a thiophene bridge†
†Electronic supplementary information (ESI) available. CCDC 1908472 and 1908473. For ESI and crystallographic data in CIF or other electronic format see DOI: 10.1039/c9sc01705d


**DOI:** 10.1039/c9sc01705d

**Published:** 2019-06-11

**Authors:** Zhihui Feng, Dandan Li, Mingzhu Zhang, Tao Shao, Yu Shen, Xiaohe Tian, Qiong Zhang, Shengli Li, Jieying Wu, Yupeng Tian

**Affiliations:** a Institutes of Physics Science and Information Technology , College of Chemistry and Chemical Engineering , Key Laboratory of Functional Inorganic Materials Chemistry of Anhui Province , Anhui Province Key Laboratory of Chemistry for Inorganic/Organic Hybrid Functionalized Materials , Anhui University , Hefei 230601 , People's Republic of China . Email: chemlidd@163.com ; Email: yptian@ahu.edu.cn; b School of Life Science , Anhui University , Hefei 230601 , P. R. China

## Abstract

Multiphoton bioimaging benefits from good penetration of tissue, low phototoxicity and high resolution. Hence, development of efficient multiphoton imaging agents is highly desirable but remains challenging. Herein, a novel terpyridine-based Zn(ii) complex bearing a thiophene bridge was designed rationally and fabricated. Thanks to its aggregation-induced emission (AIE), **DZ1** emitted bright yellow-green fluorescence (*λ*_em_ = 575 nm) under physiological conditions. The three-photon spectral changes of **DZ1** when binding with RNA unambiguously reflected its RNA-specific targeting behaviour, resulting in twofold enhancement in three-photon action cross-sections located at the second near-infrared window (1700 nm).

## Abstract 2

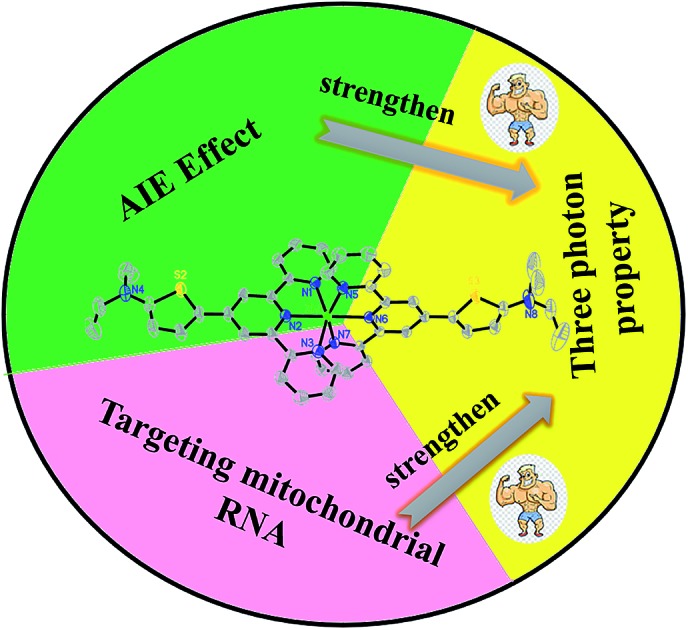
The complex **DZ1** displayed enhanced three-photon absorption activity and could avoid light-quenching and light-bleaching effectively due to its aggregation-induced emission feature.

## Introduction

Multiphoton imaging is an effective strategy because its excitation wavelength can be selected in near-infrared (NIR) optical windows and because it has high spatial resolution. With the availability of ultra-fast pulsed lasers in recent years, significant progress in three-photon absorption (3PA)-based applications has been witnessed, including three-photon pumped lasing and 3PA-based optical limiting and stabilization.^1^,^2^ Three-photon fluorescent (3PF) materials have attracted extensive attention due to their potential applications in three-dimensional optical-data storage, photodynamic therapy and biological imaging.^3^,^4^ Great efforts have been made by researchers to achieve high-3PA materials, including a series of chromophores with D–π–A–π–D, A–π–D–π–A, D–π–D and D–π–A structures.^5^–^8^ Among the strategies implemented to obtain improved 3PA responses, organic chromophores with large and extended conjugated systems for enhanced electron transfer have received increasing attention.^9^–^11^ Nevertheless, in contrast to the complicated synthesis stated above, use of metal complexes for 3PA activity, which promote electron transfer to enhance nonlinear optical (NLO) activity after coordination with metal ions, remains very rare.

Considering the applications in bioimaging, searching for NLO-active materials which utilise NIR photons as the excitation source to achieve deeper tissue penetration and weaker specimen photodamage is a highly desired target.^12^ However, most of them suffer from aggregation-caused quenching (ACQ) due to self-aggregation in physiological conditions. To meet these challenges, in contrast to ACQ, Tang and coworkers first proposed the concept of “aggregation-induced emission” (AIE).^13^ Molecules with AIE properties are more suitable for exploring 3P features because AIE-active chromophores can enhance fluorescence and 3PA action cross-sections at high concentrations.^14^,^15^ However, most of the molecules reported have been organic molecules with large conjugated systems and complicated synthetic processes. For practical applications, the search for multiphoton-active metal complexes possessing considerable fluorescence for bioimaging, which involve simple preparation, is highly desired.

**Scheme 1:**
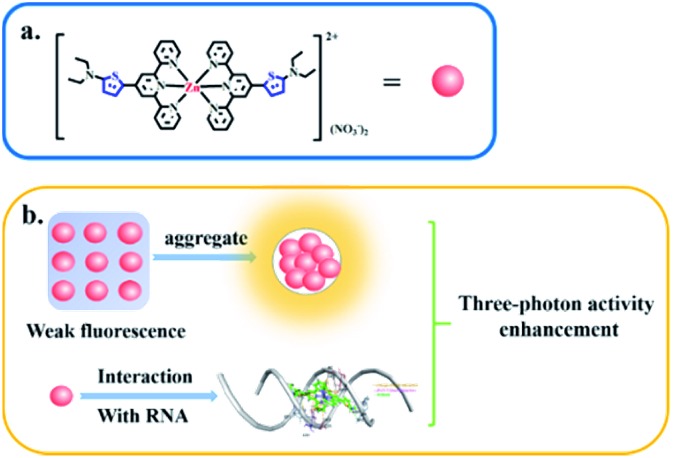
Three-photon property of complex **DZ1** in the aggregated state or upon interaction with RNA (schematic).

A novel D–A type thiophene terpyridine zinc complex (**DZ1**) was designed and synthesised. Zinc is an essential trace element in organisms. Zinc can be coordinated with terpyridine groups to obtain excellent luminescent properties and high biological activity.^16^ Thiophene is a five-membered heterocyclic compound with aromaticity, rich electron density and electronic-transmission ability. It is one of the most promising elements for designing new 3PA functional materials.^17^–^19^ Herein, we focused mainly on the three photon-absorption properties and AIE activities of DZ1 and its distinct response to RNA *in vitro*. A large 3PA cross-section (5.28 × 10^–82^ cm^6^ s^2^ per photon^2^) has been found under 1700 nm femtosecond (fs) laser excitation *via* three-photon excitation fluorescence (3PEF).^20^,^21^ DZ1 was also found to have good chemical stability, excellent biocompatibility and low toxicity, which was utilized as a multi-photon fluorescence probe for targeting mitochondria in HepG2 cells. In the future, DZ1, with its bright green-yellow AIE fluorescence and 3PA, could aid deep-tissue functional *in vivo* imaging. This is the first report on a metal–organic complex which behaves as a turn-on 3PF RNA-specific probe (Scheme 1).

## Results and discussion

### Characterization and structural features

**Fig. 1:**
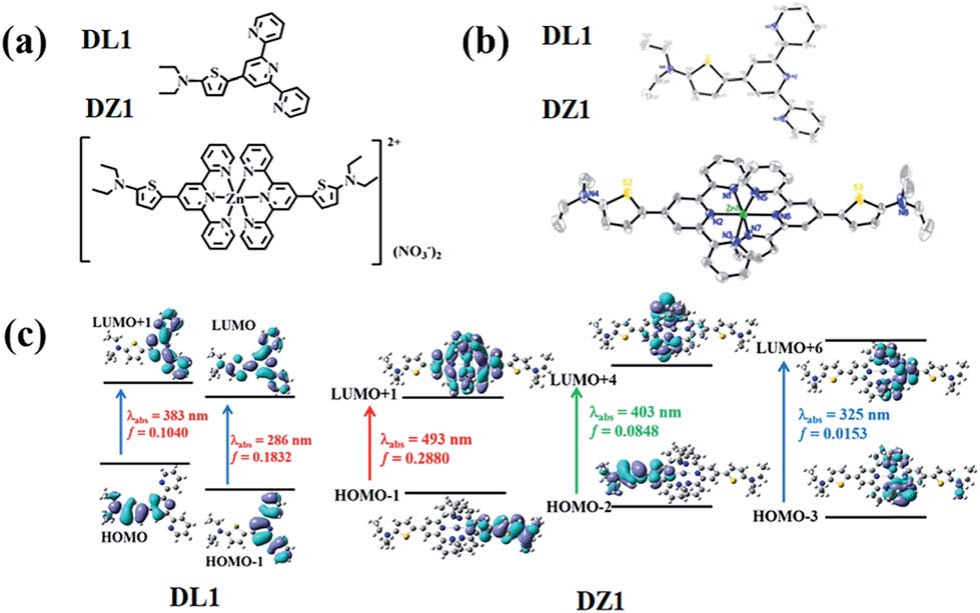
(a) Structures of **DL1** and **DZ1**. (b) Crystal structures of **DL1** and **DZ1** (H atoms and nitrate ions have been omitted for clarity). (c) Molecular orbital energy diagrams of **DL1** and **DZ1**.

The ligand **DL1** and its metal complex **DZ1** were prepared according to the synthetic routes shown in Fig. S1.† The corresponding structural features were shown in detail by single-crystal X-ray diffraction (Fig. 1a and b). **DL1** (CCDC: ; 1908472) and **DZ1** (CCDC: ; 1908473) crystallised in the triclinic crystal system with a *P*1[combining macron] space group. The terpyridine Zn complex had a classic octahedral configuration (Table S1†). The dihedral angles between the central pyridine group and thiophene ring in **DL1** and **DZ1** were 122.1° and 120.5° (**DZ1** < **DL1**), respectively. The C–C bonds between the thiophene ring and electron acceptor (pyridine ring) were 1.453 Å and 1.419 Å (**DZ1** < **DL1**), respectively (Table S2†). The coordination behaviour of the Zn(ii) centre, and the enhanced electron-withdrawing effect improve electron separation and fluidity, which favours optimised NLO activity.^22^,^23^ In accordance with theoretical calculations (Fig. 1c and S6†), a new absorption band appeared in **DZ1** compared with the ligand, which belonged to the intramolecular charge transfer (ICT) process, indicating that **DZ1** was more planar than **DL1**.

### AIE and 3PA properties

**Fig. 2:**
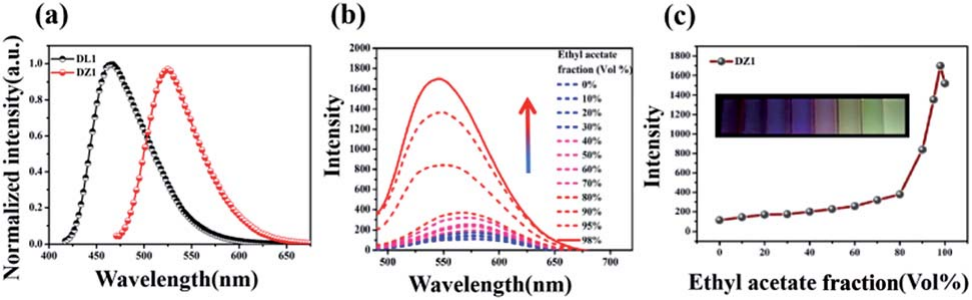
(a) Normalized fluorescence spectra for **DL1**, **DZ1** in acetonitrile (*c* = 10 μM); (b) fluorescence spectra of **DZ1** (10 μM) in ethyl acetate/acetonitrile mixtures with different ethyl acetate fractions (*λ*_ex_ = 480 nm; *λ*_em_ = 568 nm); (c) plot of emission intensity *vs.* ethyl acetate fraction, inset: photographs of the luminescence of **DZ1** in ethyl acetate fractions (0–98%) under UV illumination at 365 nm.

Encouraged by the structural features stated above, we investigated linear optical properties. The enhanced electron-withdrawing ability of complex **DZ1** gave rise to a red shift on linear fluorescence compared with the organic ligand **DL1** (Fig. 2a).^24^ In addition, in contrast to the weak fluorescence in good solvent conditions (acetonitrile), the complex **DZ1** exhibited strong fluorescence in ethyl acetate (poor solvent), which attracted our attention. We envisioned that **DZ1** had AIE in ethyl acetate/acetonitrile mixtures. To test this hypothesis, the fluorescence behaviour of **DZ1** in ethyl acetate/acetonitrile mixtures was investigated (Fig. 2b). Quantitative data revealed that, as the fraction of ethyl acetate (*f*_e_) reached 98%, the fluorescence intensity of **DZ1** was enhanced by 30-fold (Fig. 2c). The fluorescent images of the solvent mixtures at *f*_e_ = 0% to 98% in Fig. 2c (inset) presented the clear AIE effect of **DZ1**.^25^,^26^ As shown in Fig. S4,† massive C–H···π interactions and hydrogen bonds within **DZ1** were found, which could hinder the rotation of single bonds and activate restriction of intramolecular rotation (RIR), thereby resulting in AIE.^27^

**Fig. 3:**
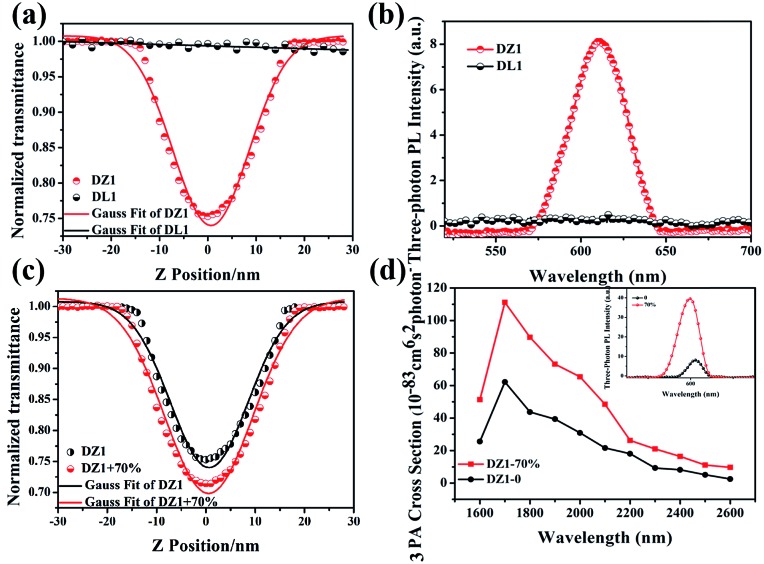
(a) Three-photon absorption spectra of **DL1** and **DZ1** (*c* = 1 mM) in DMSO obtained under an open aperture Z-scan. (b) Three-photon fluorescence spectra of **DL1** and **DZ1** (*c* = 1 mM) in DMSO; (c) three-photon absorption spectra of **DZ1** (*c* = 0.1 mM) in acetonitrile and ethyl acetate/acetonitrile (70%), obtained under an open-aperture Z-scan. (d) Changes in three-photon absorption action cross-sections of **DZ1** (*c* = 0.1 mM) in acetonitrile and ethyl acetate/acetonitrile (70%), inset: three-photon excited fluorescence spectra.

Besides, introduction of a thiophene group afforded the D–A-configurated **DZ1** with enhanced ICT behaviour, which motivated us to further evaluate its nonlinear optical property. As expected, totally different from the organic ligand **DL1** without significant NLO characteristics, **DZ1** exhibited excellent NLO behaviour including two- and 3PA activities (Fig. S6c,† 3a and b). Notably, the two- and 3PA action cross-sections of **DZ1** in the aggregated state were enhanced significantly (Fig. S6d,† 3c and d). The 3PA cross-section was calculated to be 1.11 × 10^–81^ cm^6^ s^2^ per photon^2^ by the 3PEF method (ethyl acetate/acetonitrile (70%)), which was enhanced ∼1.78-times than the molecular state (6.32 × 10^–82^ cm^6^ s^2^ per photon^2^) (Fig. 3d). The enhanced 3PA properties of **DZ1** in aggregated states were confirmed further *via* open-aperture Z-scan measurements (Fig. 3c, Table S6†).^28^ The results obtained were consistent with those of 3PEF experiments: **DZ1** unveiled an enhanced 3PA cross-section in the aggregated state. Meanwhile, a much larger 3PA cross-section could be obtained *via* the Z-scan method because it can effectively shield the energy consumed by the thermal effect and molecular rotation during photo-absorption.^29^ All the results stated above showed that **DZ1** could improve NLO activity (2PA and 3PA) based on its AIE, which was an incentive to explore further novel materials with a NLO-response.

### Cellular localization

**Fig. 4:**
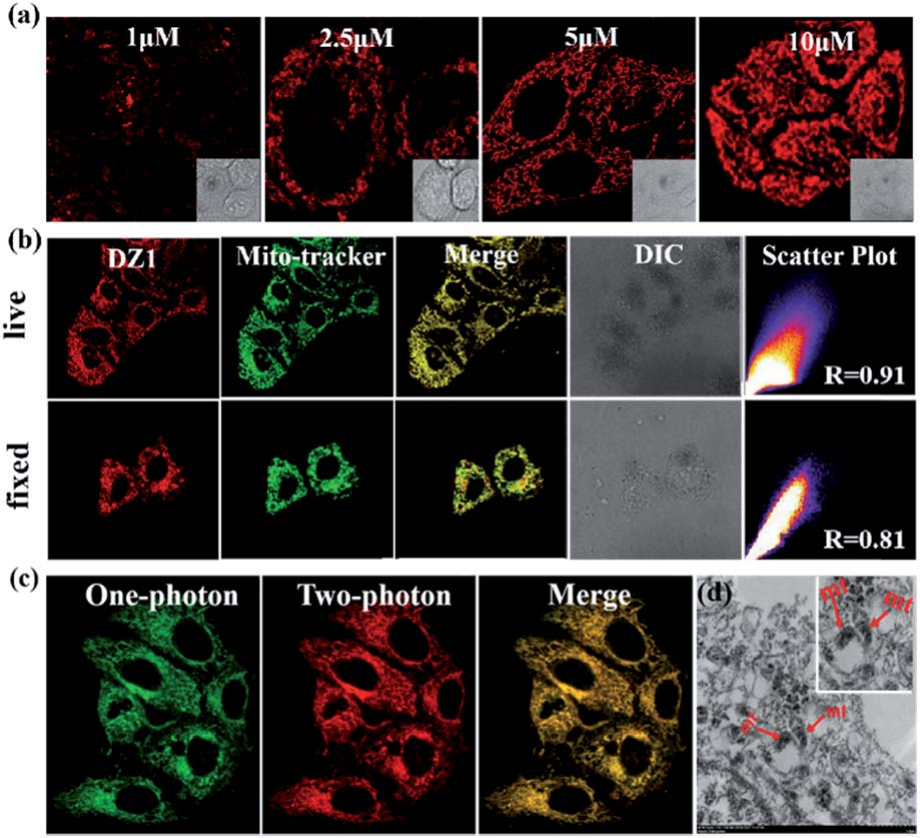
(a) Fluorescence microscopy images of HeLa cells stained with different concentrations of **DZ1** (1.0–10 μM) and in aqueous phosphate buffered saline (PBS) buffer. (b) The fluorescence development of **DZ1** in fixed cells and living cells, respectively. (c) Confocal one-photon and two-photon fluorescence microscopy image of HepG2 cells incubated with **DZ1**. (d) TEM micrograph of untreated mitochondria stained solely with **DZ1**.

Thanks to the excellent AIE and 3PA activities of **DZ1**, which can suppress ACQ behaviour in physiological environments and photodamage, the biological application of **DZ1** was investigated. As depicted in Fig. S10,† the low toxicity to cells enables **DZ1** to be a prominent fluorescent probe. To evaluate the potential biological application of probe **DZ1**, imaging experiments were carried out in HepG2 cells. First, HepG2 cells hatched for 20 min in different concentrations of **DZ1** solution.^30^ We found that **DZ1** passed through the cell membrane and stained mitochondria with an optimum concentration of 10 μM (Fig. 4a). A co-localization experiment was done using the MitoTracker™ (a green fluorescent protein that targets mitochondria). The stained cells emitted red fluorescence from **DZ1** and green fluorescence from MitoTracker (Fig. 4b). The merged image showed that the distribution of **DZ1** in cells was consistent with that of MitoTracker, indicating the high selectivity of **DZ1** towards mitochondria.^31^ Also, it showed that **DZ1** could locate firmly in mitochondria in living and fixed cells (Fig. 4b), indicating that **DZ1** is a mitochondrial membrane potential-independent mitochondrial probe. As reported,^32^,^33^ the hydrophobicity of cationic probes plays a vital part in mitochondria staining. Therefore, the hydrophobicity (as modelled by log *P* values) of **DZ1** was measured (log *P* of **DZ1** = 0.489), which corroborated the moderate hydrophobicity of **DZ1**. In addition, the log *P* of **DZ1** was in the reported range (0–5), which further demonstrated its specific targeting effect on mitochondria.^34^ Hence, the hydrophobic cation of **DZ1** could be used to target mitochondria.

Because of its remarkable advantages of increased penetration depth, low tissue autofluorescence, and reduced photodamage, two-photon fluorescence microscopy has drawn considerable attention in non-destructive imaging.^35^ Bio-imaging studies of **DZ1** were carried out using confocal microscopy with a two-photon (2P) excitation wavelength at 760 nm and one-photon (1P) at 405 nm (emission = 450–600 nm), respectively. As shown in Fig. 4c, compound **DZ1** could penetrate cell membranes and distribute throughout the cytosolic region from 1P- and 2P channels. All experiments showed that **DZ1** could be employed as a two-photon fluorescent (2PF) probe to mark mitochondria in cells. Besides the 2P microscopic imaging results, transmission electron microscopy was used to determine the **DZ1** distribution in cells.^36^ According to Fig. 4d, **DZ1**-labelled cells showed bright ridges and internal moments on mitochondria, indicating that most zinc substances were present in the mitochondria. To clarify indirectly the advantages of 3PF imaging, we undertook 1PF and 2PF tissue imaging. As shown in Fig. S11,† the 1PF (*λ*_ex_ = 405 nm) and 2PF (*λ*_ex_ = 780 nm) imaging performance of **DZ1** in fixed mouse cardiac tissue was evaluated. Obviously, the 2PF signals with high contrast could be detected at a depth ≤54 μm for **DZ1** (the detection depth of 1PF was 30 μm). These results revealed that a deeper penetration depth could be obtained by 2PF imaging. These results implied that **DZ1** could be employed for 3PF imaging under excitation of NIR-II light with very deeper penetration depth.^37^

### Studies on RNA binding

**Fig. 5:**
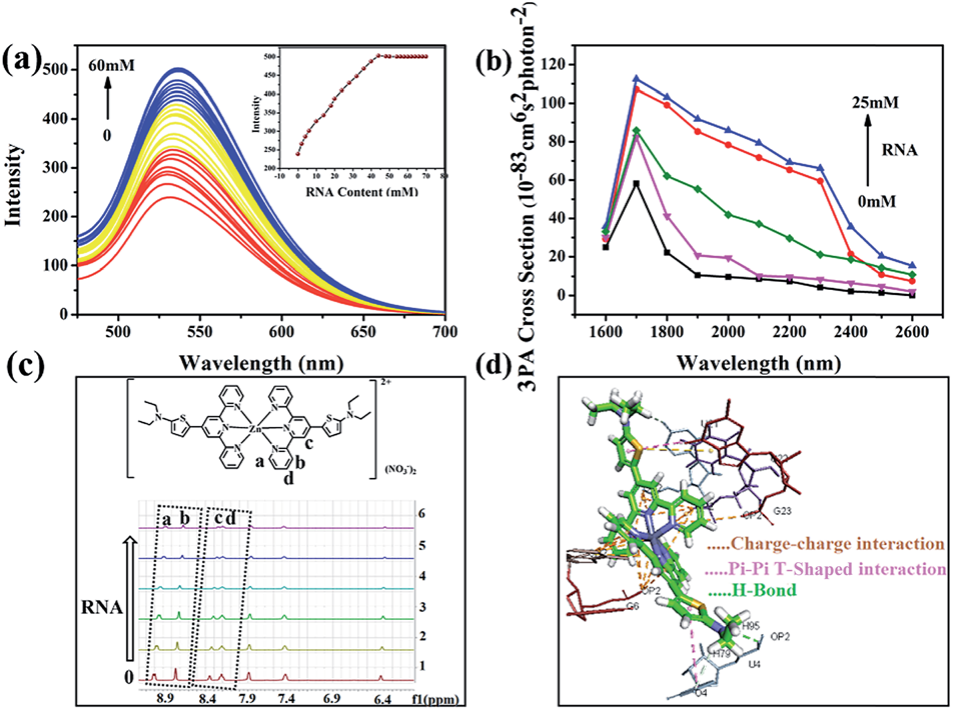
(a) Fluorescence intensity of **DZ1** (10 μM) under various amounts of RNA (0–60 mM) in Tris–HCl buffer (pH = 7.4), inset: limit point diagram of RNA titration; (b) three-photon absorption action cross-section changes of 1 mM **DZ1** with 25 mM RNA. (c) Partial ^1^H-NMR titration spectra for **DZ1** + RNA in D_2_O. (d) Models obtained after molecular modeling for the interaction of **DZ1** with RNA fragment.

To obtain more insight into how **DZ1** targets mitochondria, various amino acids and proteins were screened *in vitro*. Obviously, **DZ1** showed a distinct response to RNA (Fig. S12a†). The interaction of **DZ1** with RNA (mitochondria) was tested preliminarily by 1PF (Fig. 5a), 3PF (Fig. S12c†) and 3PA spectroscopy (Fig. S12d†).^38^ As shown in Fig. 5a, **DZ1** (10 μM) was titrated by adding different concentrations of RNA in Tris–HCl buffer (pH = 7.4). The fluorescence intensity reached a maximum and maintained an equilibrium when the amount of RNA reached 44 mM, and the fluorescence intensity increased by 2.1-times compared with that in pure dimethyl sulfoxide (DMSO).^39^,^40^ Given that there was weak fluorescence emission for **DZ1** in DMSO, the NLO properties of **DZ1** with RNA were also measured by open-aperture Z-scanning and 3PF. When RNA was added, the 3PA cross-section (*σ*) increased obviously (Fig. 5b, Table S6†). The 3PA cross-section increased 1.92-fold (*σ* = 1.17 × 10^–81^ cm^6^ s^2^ per photon^2^ at 1700 nm) after 25 mM RNA was added into the system; this may have been due to the interaction between **DZ1** and RNA.^41^ To confirm further that **DZ1** was internalised with RNA, ^1^H-NMR titration experiments were carried out in D_2_O. Fig. 5c shows that the proton signals Ha, Hb, Hc and Hd on the pyridine ring moved towards high magnetic fields after RNA addition, with the signal intensity decreasing gradually. These data indicated that the location of hydrogen atoms may be the binding sites of RNA.^42^ Molecular-docking simulation showed that the interaction between the **DZ1** molecule and RNA was a charge–charge interaction because the RNA surface was anionic and **DZ1** possessed a positive charge (Fig. 5d). As revealed by Horobin and coworkers,^34^ only probes with moderate hydrophobicity will penetrate mitochondrial membranes passively. Therefore, moderate hydrophobicity enabled **DZ1** to penetrate passively through mitochondrial membranes and then recognise mitochondrial RNA specifically by charge action. Considering the results mentioned above, we suggest tentatively that **DZ1** can target mitochondria due to its hydrophobic cation, and then bind mitochondrial RNA through electrostatic actions.

## Conclusions

We synthesised a novel AIE-active metal complex **DZ1** that could be used to label mitochondria in live and dead cells with distinct multi-photon fluorescence under fs laser excitation. In addition, **DZ1** showed an excellent response to RNA, which had a large 3PA action cross-section of 1.17 × 10^–81^ cm^6^ s^2^ per photon^2^. In addition, compared with commercially multiphoton probes, AIE-active probe **DZ1** suppressed ACQ behaviour due to self-aggregation under physiological conditions, but was also synthesised simply and exhibited excellent photo-stability. The unique 3PA of the AIE-active complex may find potential high-tech applications in biomedical theranostics and photonic devices.

## Conflicts of interest

There are no conflicts to declare.

## Supplementary Material

Supplementary information

Crystal structure data
