# Supplementary material for: Enhanced three-photon activity triggered by the AIE behaviour of a novel terpyridine-based Zn(ii) complex bearing a thiophene bridge
Source: Chem Sci. 2019 Jun 11;10(30):7228–32. doi: 10.1039/c9sc01705d (PMC6677111; doi:10.1039/c9sc01705d)
Supplement: Supplementary file 1 [file SC-010-C9SC01705D-s001.pdf]

Electronic Supplementary Material (ESI) for Chemical Science.
This journal is © The Royal Society of Chemistry 2019

Supporting Information

Enhanced three-photon activity trigged by AIE behavior of a novel terpyridine-based Zn(II) complex bearing thiophene bridge

Zhihui Feng[a], Dandan Li[a*], Mingzhu Zhang[a], Tao Shao[a], Yu Shen[a], Xiaohe Tian[b], Qiong Zhang[a], Shengli Li[a], Jieying Wu[a], Yupeng Tian[a*].

[a] Institutes of Physics Science and Information Technology, College of Chemistry and Chemical Engineering, Key Laboratory of Functional Inorganic Materials Chemistry of Anhui Province, Anhui Province Key Laboratory of Chemistry for Inorganic/Organic Hybrid Functionalized Materials, Anhui University, Hefei 230601, People's Republic of China Institutes of Physics Science and Information Technology, Anhui University, Hefei 230601, China

[b] School of Life Science, Anhui University, Hefei 230601, P. R. China

[*]Corresponding author: chemlidd@163.com; yptian@ahu.edu.cn

Materials and apparatus....4
Experimental section....4
1.1 Synthesis and characterization....4
Fig. S1. Synthetic routes for **DL1** and **DZ1**....4
1.2 Crystallography....5
1.3 Computational details....5
1.4 Two-photon Z-scan measurements....6
1.5 Three-photon excitation fluorescence (3PEF) measurements....6
1.6 Three-photon Z-scan measurements....7
1.7 Molecular docking with RNA....7
1.8 Cell imaging....8
Fig. S2. MALDI-TOF mass spectrum of **DL1**....8
Fig. S3. ^{1}H NMR spectrum of **DL1**....9
Fig. S4. MALDI-TOF mass spectrum of **DZ1**....9
Fig. S5. ^{1}H NMR spectrum of **DZ1**....10
Fig. S6. UV-*vis* absorption spectra of compounds **DL1** and **DZ1** in different solvents (c = 10 μM)....10
Fig. S7. One-photon excited fluorescence spectra of compounds **DL1** and **DZ1** in different solvents (c = 10 μM)....10
Fig. S8. The interactions in crystal structures of **DL1**, **DZ1**....11
Fig. S9. (a) Absorption spectra of complexes **DZ1**(c = 10 μM) in ethyl acetate/acetonitrile mixtures with different ethyl acetate fractions (0–98 %); (b) DLS measurements of **DZ1** dispersions in ethyl acetate/acetonitrile (*f*e = 95 %) mixtures at 20 μM, (inset) SEM pictures of ethyl acetate/acetonitrile (*f*e = 95 %) dispersions of **DZ1** at 20 μM. (c) Two-photon absorption spectra of **DL1** and **DZ1** (c = 1 mM) in DMSO, obtained under an open aperture Z-scan; (d) Two-photon absorption spectra of **DZ1** (c = 0.1 mM) in acetonitrile and ethyl acetate/acetonitrile (70 %), obtained under an open aperture Z-scan....11
Fig. S10. Cytotoxicity data results obtained from the MTT assay....12
Fig. S11. Ex vivo (a) one- (λ_{ex} = 405 nm) and (b) two-photon (λ_{ex} = 780 nm)

microscopy images of Mouse cardiac tissue stained with 0.1 μM **DZ1** at different penetration depths along the Z axis...12
Fig. S12. (a) The fluorescence spectra changes of 10 μM **DZ1** with different amino acid, X represent all common amino acids (Ala, Arg, Asp, Cys, Gln, Glu, His, Ile, Gly, Asn, Leu, Lys, Met, Phe, Pro, Ser, Thr, Trp, Tyr and Val), histone, DNA, RNA; (b) Three-photon fluorescence intensity of **DZ1** (1mM) under various amounts of RNA (0–25 mM) in Tris–HCl buffer (pH = 7.4); (c) Three-photon absorption action cross-section changes of 1mM **DZ1** with 20 mM RNA 13
Fig. S13. Schematic diagram of the binding of the **DZ1** molecule to the RNA chain.13
Table S1. Crystal data and structure refinement for **DL1** and **DZ2**............................14
Table S2. Selected bond lengths (Å) and angles (°) for the crystal structure of **DL1** and **DZ1**..14
Table S3. Selected experimental and computed optical data 15
Table S4. Single-photon photophysical properties of **DL1** and **DZ1** in different solvents…………………………………………………………………………16
Table S5. Two-photon absorption data for **DZ1** 16
Table S6. Three-photon absorption data for **DZ1** 16
References 17

Materials and Apparatus

All chemicals were commercially available and used without further purification; the solvents were of analytical grade. The ^{1}H-NMR and ^{13}C-NMR spectra recorded at 25℃ using a Bruker 400 Ultrashield were reported as parts per million (ppm) from TMS (δ). IR spectra were recorded on a Nicolet FTIR-instrument. MALDI-TOF mass spectra were recorded using Bruker Autoflex III Smartbeam. ESI Mass Spectrometer was recorded using LCQ Fleet. UV-vis absorption spectra were recorded on a UV-265 spectrophotometer. Fluorescence measurements were carried out on a Hitachi F-7000 fluorescence spectrophotometer. The concentration of the sample solution was 1.0×10^{-5} mol/L.

Experimental Section

1.1 Synthesis and characterization

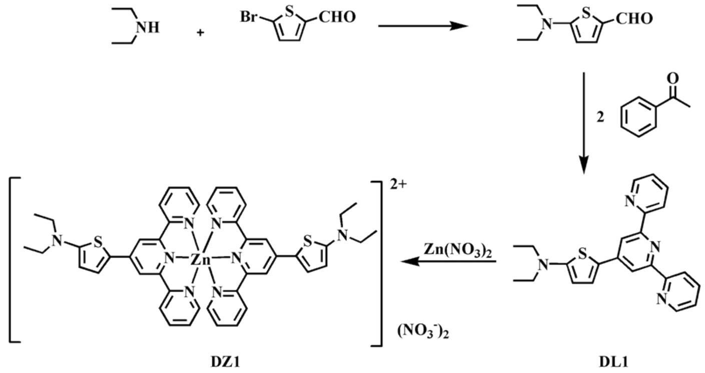

Fig. S1. Synthetic routes for **DL1** and **DZ1**

DL1: 5-N,N-diethylthiophene-2-formaldehyde (1.83g, 0.01mol) and 2-acetyl pyridine (2.44 g, 0.02 mol) were stirred in 50 mL ethanol. Ethanol dissolves 3.2 g KOH and slowly drops into the reaction liquid, heating to 65°C, reaction 6 h. Steamed out part of ethanol, and the supernatant was poured out, obtained red sticky products, with a small amount of ethanol for ultrasound, there are solid precipitates, suction filtration, get red solid. Recrystallization of ethanol to obtain products. Yield 71 %. ^{1}H NMR (400 MHz, d_6- DMSO, ppm): δ 8.75 (d, J = 4.2 Hz, 2H), 8.61 (d, J = 7.9 Hz, 2H), 8.40 (s, 2H), 8.00 (t, J = 8.4 Hz, 2H), 7.67 (t, J = 6.4 Hz, 1H), 7.57 - 7.47 (m,

2H), 6.00 (d, J = 4.2 Hz, 1H), 3.41 (q, J = 7.1 Hz, 4H), 1.19 (t, J = 7.0 Hz, 6H). ^{13}C NMR (100 MHz, d_6- DMSO, ppm): δ 158.6, 155.1, 149.2, 143.7 137.3, 128.2, 124.3, 120.7, 113.4, 102.2, 46.6, 12.1. FT-IR (KBr, cm^{-1}): 3037 (m), 1932 (vw), 1865 (w), 1791 (m), 1582 (s), 1464 (s), 1006 (s), 792 (s), 693 (s). MALDI-TOF: m/z, cal.: 386.2, found: 387.2 $[M+1]^+$.

DZ1: The ligand **DL1** (0.76 g, 2.0 mmol) was dissolved in methanol, and $Zn(NO_3)_2$ **.**$6H_2O$ (0.29g, 1.0mmol) was dissolved in methanol and added to the above solution. This mixture was heated to reflux for 3 h, stop reaction. Most of the methanol was evaporated and cooled to room temperature, and a large number of red microcrystals are gradually precipitated. Filtration with less methanol washed, dried, get the complex **DZ1**. Yield 93 %. ^{1}H NMR (400 MHz, d_6- DMSO, ppm): δ 9.11-8.89 (m, 4H), 8.75 (s, 4H), 8.36 (d, 4.1 Hz, 2H), 8.21 (t, J = 7.8 Hz, 4H), 7.89 (d, J = 4.8Hz, 4H), 7.58 – 7.33 (m, 4H), 6.34 (d, J = 4.2 Hz, 2H), 3.54 (d, J = 6.9 Hz, 8H), 1.28 (t, J = 7.0 Hz, 12H). ^{13}C NMR (100 MHz, d_6-DMSO, ppm): δ 162.7, 148.3, 147.8, 146.9, 141.0, 127.1, 122.6, 117.9, 114.6, 103.9, 47.1, 11.9. FT-IR (KBr, cm^{-1}): 3418, 2917, 1600, 1572, 1548, 1488, 1473, 1439, 1378, 1224,1159, 1075, 1022, 896, 790, 748, 698, 640, 517. MALDI-TOF-MS: m/z, cal.: 960.24, found: 419.25 $[M-2NO_3^-]^{2+}/2$.

1.2 Crystallography

The single crystal X-ray diffraction measurements were performed on Bruker Smart 1000 CCD area detector diffractometer. The determination of unit cell parameters and data collections were performed with Mo-K_α (λ = 0.71073 Å). And all structures were solved by direct methods and difference Fourier syntheses. The non-hydrogen atoms were refined anisotropically and hydrogen atoms were introduced geometrically. The final refinement was performed with SHELXTL-97 program package. To deeply understand the relationship between molecule geometry and optical property, we tried to obtain as many as single-crystal structures of the compounds.

1.3 Computational details

Optimization was performed on B3LYP [LANL2DZ] without any symmetry

restraints, and the TD-DFT {B3LYP[LANL2DZ]} calculations were carried out with the crystal structure or the optimized structure. All calculations, including optimization and TD-DFT, were performed using the G09 software. Geometry optimization of singlet-singlet excitation energies were carried out with a basis set composed of 6-31G(d) for C N S O H atoms, and the LANL2DZ basis set for Zn atoms. The basis set was downloaded from the EMSL basis set library. The lowest 25 spin-allowed singlet-singlet transitions were taken into account in the calculation of the absorption spectra.

1.4 Two-photon Z-scan measurements

To study the third-order NLO properties of **DL1** and **DZ1**, their nonlinear 2PA coefficient (β) and the molecular 2PA cross-sections (σ) were obtained by an open-aperture Z-scan technique using a femtosecond laser pulse (680-1080 nm, 80 MHz). The pulse length was 140 fs, the thermal heating of the sample with high repetition rate laser pulse was removed by the use of a mechanical chopper running at 10 Hz, and the average laser power was 36 mW. The sample in DMSO (its thickness is 1 mm) was put in the light path, and all measurements were carried out at room temperature. The filled squares represent the experimental data, and the solid line is the theoretical curve modified from the following equations:

$$T(Z,S=1)=\sum_{m=0}^{\infty}\frac{-q_0(Z)^m}{(m+1)^{3/2}}$$

$$q_0(Z)=\frac{\beta\cdot I_0\cdot L_{eff}}{1+x^2}$$

where $x = z/z_0$, in which $z_0 = \pi\omega_0^2/\lambda$ is the diffraction length of the beam, where ω_0 is the spot size at the focus, λ is the wavelength of the beam, and z is the sample position. I_0 is the input intensity at the focus $z = 0$ and equals the input energy divided by $\pi\omega_0^2$. $L_{eff} = (1 - e^{-\alpha L})/\alpha$ is the effective length, in which α is the linear absorption coefficient and L is the sample length.

1.5 Three-photon excitation fluorescence (3PEF) measurements

All the samples were contained in 1cm-optical length quartz liquid cell and

Rhodamin 6G as the standard sample for intensity comparison. Spectrometer: Ocean Optics QE65 Pro (300-2500 nm). Laser: Coherent Astrella + TOPAS Prime (1600-2600 nm, 1 kHz, 120 fs. All the results provided here are the 10 times average results.[1]

Theory:

$$\sigma_{(S)} = \frac{C_R \cdot n_S \cdot F_S \cdot Q_R}{C_S \cdot n_R \cdot \mathrm{F}_R \cdot Q_S} \cdot \sigma^3{}_{(R)}$$

Here, the subscript R stands for the reference molecule, S is the sample molecules, σ is the 3PA cross-section value, C is the concentration of the solution, n is the refractive index of the solution, F is the integrated area of the detected three-photon-induced fluorescence signal, and Q is the fluorescence quantum yield.

1.6 Three-photon Z-scan measurements

The laser pulses were produced by Laser: Coherent Chameleon Ultra II+OPO (1100-1700 nm, 1 kHz, 140 fs, which focused onto a 1-cm-thick quartz cuvette containing the solutions of the derivatives. The incident and transmitted laser pulse energy were monitored by moving the cuvette along the propagation direction of the laser pulses. Z-scans display a symmetric valley with respect to the focus, typical of an induced positive NLA effect. By fitting the trances of Z-scan theories by three-photon theory, we obtain the nonlinear absorption coefficient γ (3PA) at different levels of I_0.[2]

Theory:

$$\gamma = \frac{{I_0}^2 / I^2 - 1}{2 \times L \times {I_0}^2}$$

$$\sigma = \frac{\gamma}{N_A \times d_0 \times 10^{-3}} \bullet \left(\frac{hc}{\lambda} \right)^2$$

Here, I_0 is the optical power density, and I is the outgoing light intensity, L is the sample cell thickness, γ is the three-photon absorption coefficient (cm^3/W^2), d_0 is the sample concentration, λ is the wavelength, h is Planck's constant ($6.626*10^{-34}$ J·s), N_A is the Avogadro constant ($6.022*10^{23}$ mol^{-1}).

1.7 Molecular docking with RNA

The structure of RNA (5'GAGUAGAAACAAGGCUU/GAAGCCUUGUUUCUACUC'3) was establishment by nuclear building of software. The structure of RNA is processed. The active-site cavities of RNA is defined using the biggest cavity of the surface of RNA. Docking was carried out using the docking method of CDOCK. All calculation were performed by Discovery Studio.

1.8 Cell imaging

Cells were seeded in 24 glass bottom well plates at a density of $1x10^4$ cells per well and grown for 96 hours. For live cell imaging, the cells were incubated for 30 min in DMEM culture medium supplemented with 10 % FCS, L-glutamine and Fungizone at 37 °C in an atmosphere of 5 % CO_2 and 95 % air. The cells were then washed with PBS (3 x 1 mL per well) and 1 mL of PBS was added to each well. The cells were imaged using a confocal laser scanning microscope.

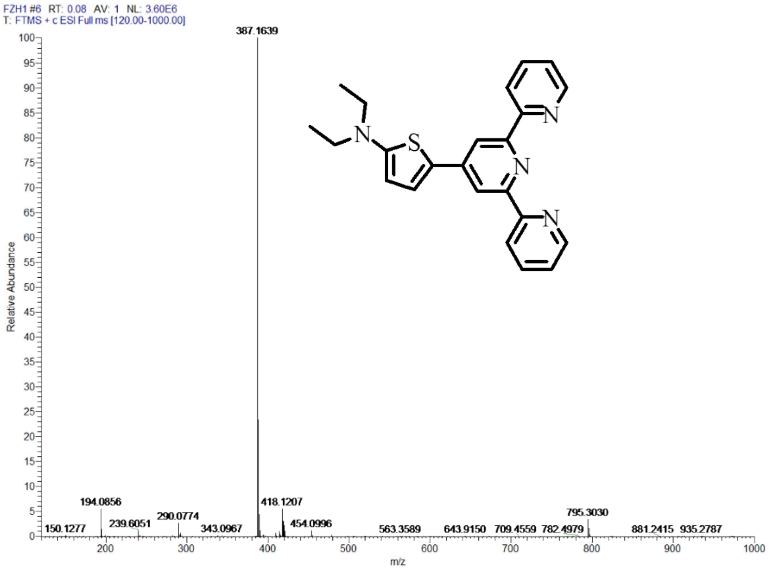

Fig. S2. MALDI-TOF mass spectrum of **DL1**

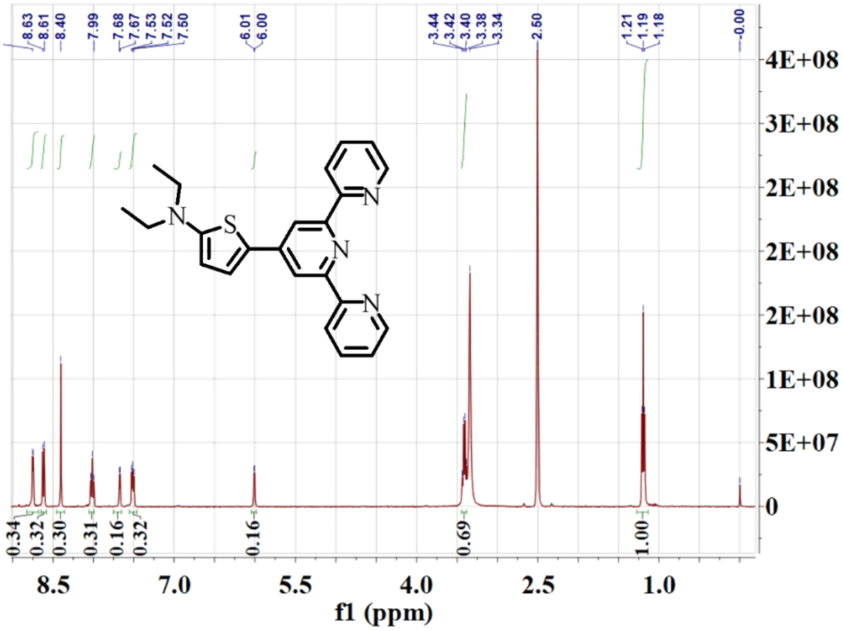

Fig. S3. ^{1}H NMR spectrum of **DL1**

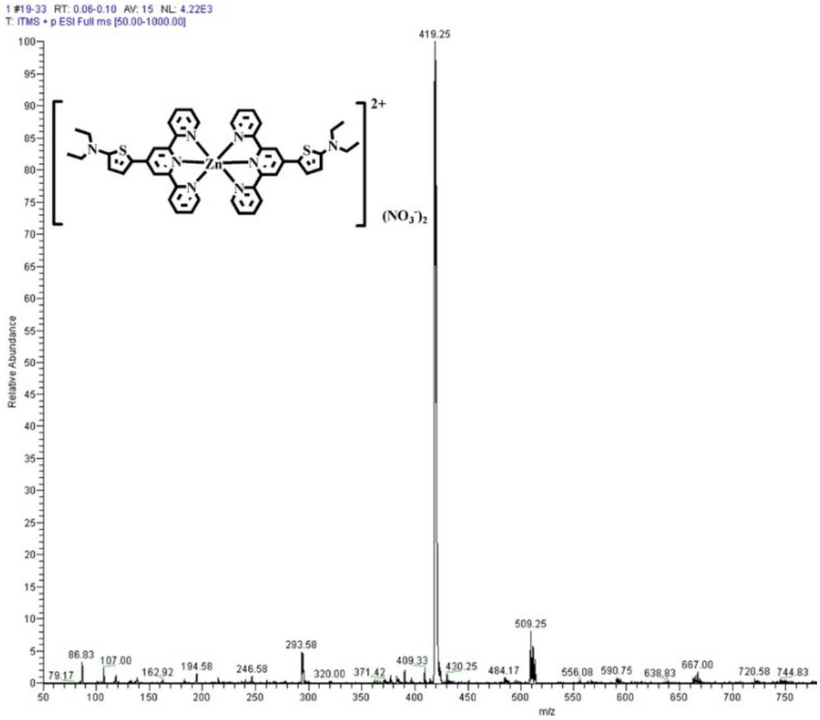

Fig. S4. MALDI-TOF mass spectrum of **DZ1**

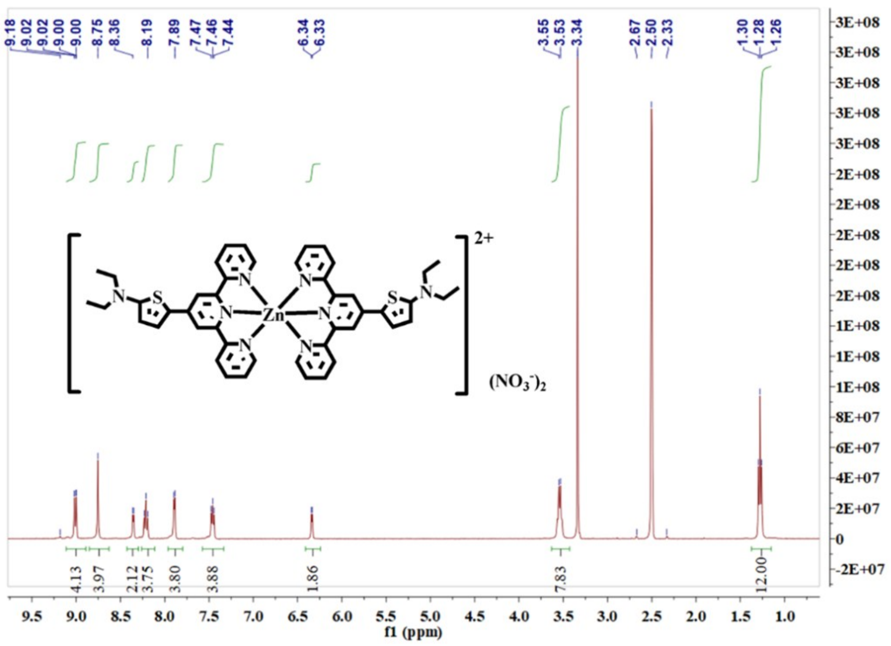

Fig. S5. ^{1}H NMR spectrum of **DZ1**

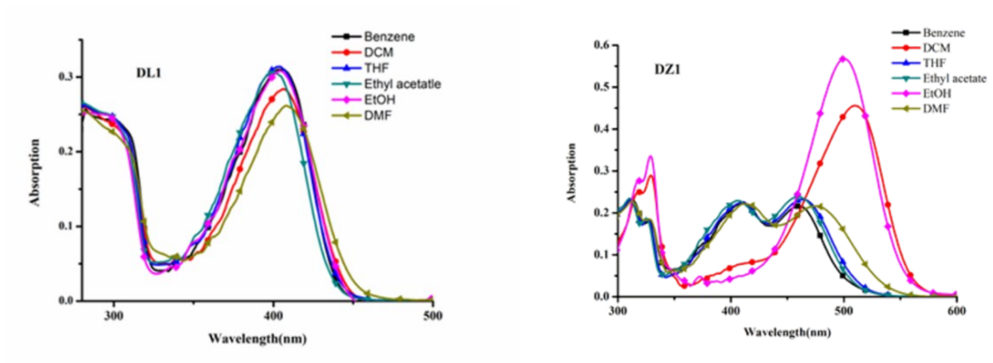

Fig. S6. UV-*vis* absorption spectra of ligand **DL1** and complex **DZ1** in different solvents (c = 10 μM).

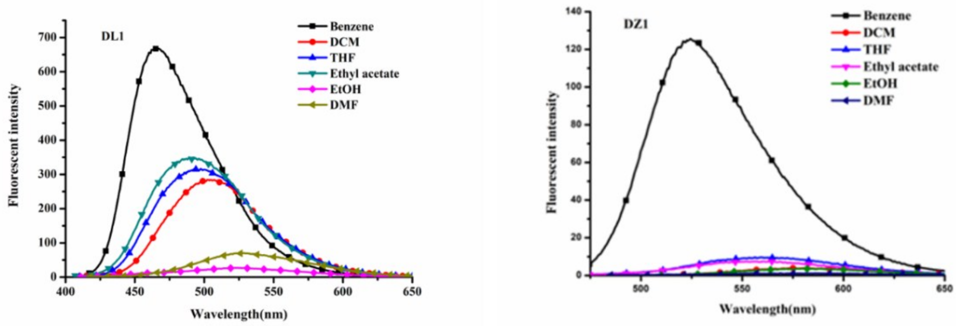

Fig. S7. One-photon excited fluorescence spectra of ligand **DL1** and complex **DZ1**in different solvents (c = 10 μM).

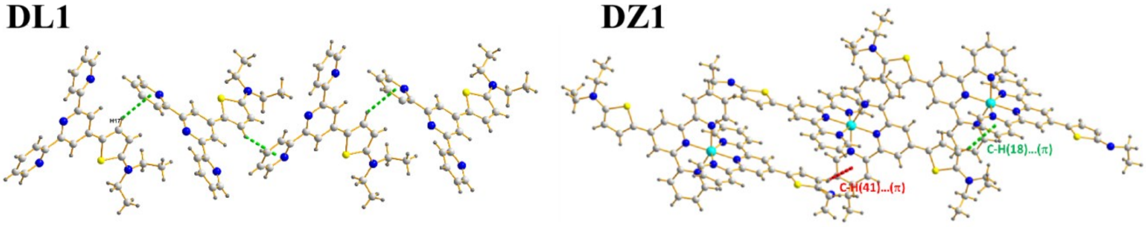

Fig. S8. The interactions in crystal structure of **DL1, DZ1**

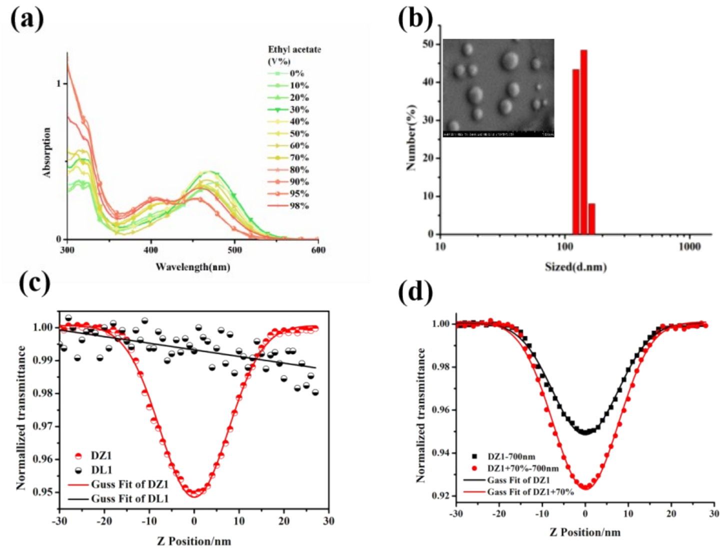

Fig. S9. (a) Absorption spectra of complex **DZ1**(c = 10 μM) in ethyl acetate/acetonitrile mixtures with different ehyl acetate fractions (0–98 %); (b) DLS measurements of **DZ1** dispersions in ethyl acetate/acetonitrile (*f*e = 95 %) mixtures at 20 μM, (inset) SEM pictures of ethyl acetate/acetonitrile (*f*e = 95 %) dispersions of **DZ1** at 20 μM; (c) Two-photon absorption spectra of **DL1** and **DZ1** (c = 1 mM) in DMSO, obtained under an open aperture Z-scan; (d) Two-photon absorption spectra of **DZ1** (c = 0.1 mM) in acetonitrile and ethyl acetate/acetonitrile (70 %), obtained under an open aperture Z-scan.

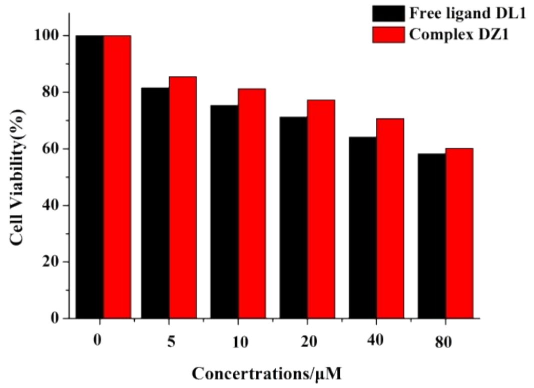

Fig. S10. Cytotoxicity data results obtained from the MTT assay.

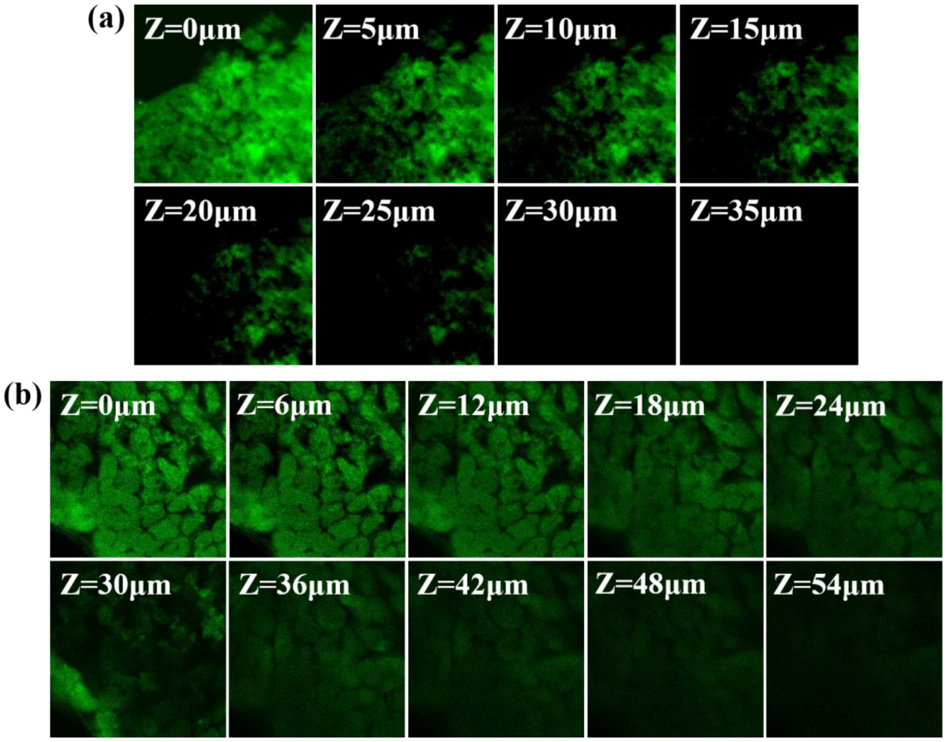

Fig.S11 Ex vivo (a) one- (λ_{ex} = 405 nm) and (b) two-photon (λ_{ex} = 780 nm) microscopy images of Mouse cardiac tissue stained with 0.1 μM **DZ1** at different penetration depths along the Z axis.

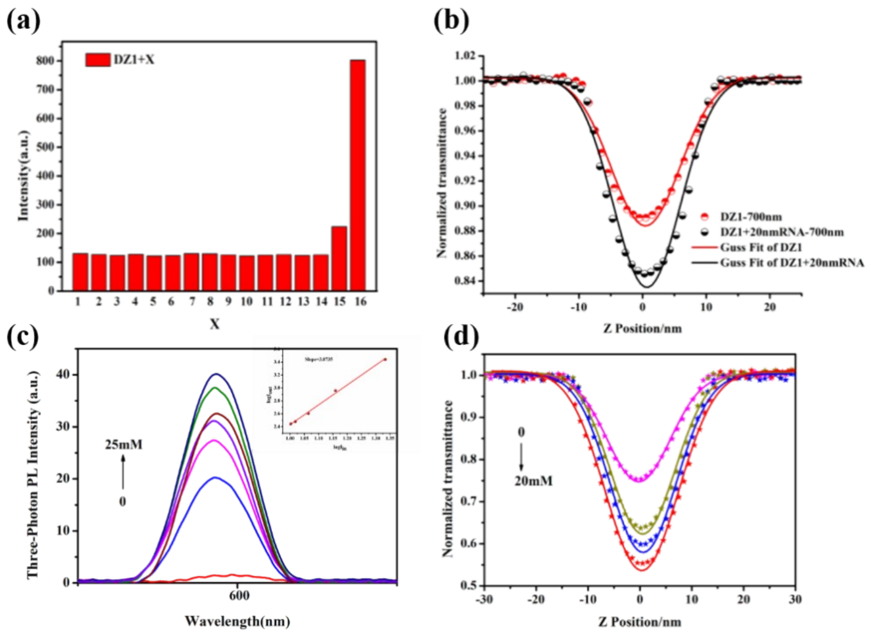

Fig. S12. (a) The fluorescence spectra changes of 10 μM **DZ1** with different amino acid, X represent all common amino acids (Ala, Arg, Asp, Cys, Gln, Glu, His, Ile, Gly, Asn, Leu, Lys, Met, Phe, Pro, Ser, Thr, Trp, Tyr and Val), histone, DNA, RNA; (b) Two-photon absorption spectra of **DZ1** (c = 0.1 mM) in DMSO and after the addition of 20 mM RNA, obtained under an open aperture Z-scan. (c) Three-photon fluorescence intensity of **DZ1** (1 mM) under various amounts of RNA (0-25 mM) in Tris–HCl buffer (pH = 7.4), inset: Three-photon verification in **DZ1** (1 mM) when 25mM RNA was added; (d) Three-photon absorption spectra changes of 1 mM **DZ1** with 20 mM RNA.

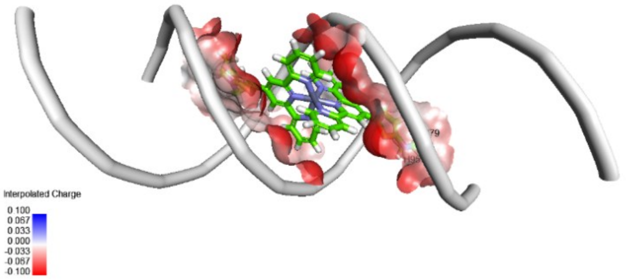

Fig. S13. Schematic diagram of the binding of the **DZ1** molecule to the RNA chain.

Table S1. Crystal data and structure refinement for **DL1** and **DZ2**.

**Ligand**	**DL1**	**DZ1**
Empirical formula	$C_{23}H_{22}N_4S$	$C_{46}H_{44}N_{10}O_6S_2Zn$
Formula weight	386.51	962.4
Temperature	293(2) K	296(2) K
Wavelength	0.71073	0.71073
Crystal system	Triclinic	Triclinic
Space group	$P\bar{1}$	$P\bar{1}$
Unit cell dimensions	a = 6.149(2)Å	a = 13.075(2)Å
	b = 19.493(7) Å	b = 13.076(2) Å
	c = 17.804(6) Å	c = 15.476(3) Å
	a = 85.124(2)°, β= 110.205(2)°, g= 95.105(2)°	a = 99.480(2), β= 95.071(2)°,g= 105.389(2)°
Volume	2002.7(12) $Å^3$	2491.7(7) $Å^3$
Z, Calculated density	4, 1.282 Mg/m^3	2, 1.283 Mg/m^3
Absorption coefficient	0.177 mm^{-1}	0.632 mm^{-1}
F(000)	816	1000
Crystal size	0.30 □ 0.20□ 0.20 mm	0.30 □ 0.20□ 0.20 mm
Theta range for data collection	2.09 to 25.00°	1.63 to 25.00°
Limiting indices	-7<=h<=7, -23<=k<=23, -21<=l<=20	-15<=h<=15, -14<=k<=15, -18<=l<=18
Reflections collected / unique	9028 / 2445 [R(int) = 0.0356]	17642 / 8665 [R(int) = 0.0254]
Max. and min. transmission	0.9671 and 0.9620	0.8840 and 0.8331
Data / restraints / parameters	2445 / 0 / 255	8665 / 1 / 590
Goodness-of-fit on F^2	1.007	1.093
Final R indices	R_1 = 0.0520, wR_2 = 0.1450	R_1 = 0.0684, wR_2 = 0.2171
Largest diff. peak and hole	0.201 and -0.135 e. $Å^{-3}$	1.358 and -0.638 e. $Å^{-3}$

Table S2. Selected bond lengths (Å) and angles (°) for the crystal structure of **DL1** and **DZ1**

**DL1**	Dist.	**DZ1**	Dist
N1-C1	1.344(4)	Zn1-N1	2.183(3)
N1-C5	1.340(4)	Zn1-N2	2.057(3)
N2-C6	1.340(4)	Zn1-N3	2.184(3)
N2-C10	1.350(4)	C8-C16	1.419(6)
N3-C11	1.335(4)	S2-C16	1.755(5)
N3-C15	1.352(4)	S2-C19	1.746(5)

N4-C19	1.371(4)	N4-C19	1.399(7)
N4-C20	1.454(5)	N4-C20	1.465(8)
C16-C8	1.453(4)	N4-C22	1.419(9)
	Angle(°)		Angle(°)
N2-C6-C5	115.6(2)	N1-Zn1-N2	76.05(12)
N2-C6-C7	123.6(3)	N2-Zn1-N3	75.53(13)
N2-C10-C11	116.4(3)	N2-Zn1-N6	179.23(13)
N4-C19-C18	128.6(3)	N6-Zn1-N7	75.59(12)
C20-N4-C22	120.2(3)	N6-Zn1-N1	103.76(12)
C5-N1-C1	117.4(3)	N7-Zn1-N3	92.21(13)
C11-N3-C15	116.2(3)	N3-Zn1-N5	97.18(13)
C9-C8-C7	116.3(3)	N7-Zn1-N5	151.16(13)
C7-C8-C16	123.1(3)	C8-C16-S2	120.5(4)
C8-C16-C17	129.1(3)	C8-C16-C17	131.8(5)
C9-C8-C16	120.6(3)	C16-C8-C9	120.6(2)
S1-C16-C8	122.1(2)	S1-C16-C8	122.1(2)

Table S3. Selected experimental and computed optical data

**Compd.**	**OI**[a]	**ΔE(eV)**[b]	**Cal.λ_{max} (nm)**[c]	**Obs.λ_{max} (nm)**[d]	**f**[e]	**Character**
**DL1**	H→L+1	3.2409	383	396	0.1040	ICT
	H-1→L	4.3254	286	291	0.1832	π→π*
**DZ1**	H-1→L+1	3.3572	493	495	0.2880	ICT (LLCT)
	H-2→L+4	3.7569	403	397	0.0848	ICT (LLCT)
	H-3→L+6	3.9143	325	321	0.0153	π→π*

[a] Orbitals involved in the excitations.

[b] Excitation energies (eV).

[c] Calculated peak position of the longest absorption band in ethanol.

[d] Observed peak position of the longest absorption band in ethanol.

[e] Oscillator Strengths, H represents HOMO, L represents LUMO.

Table S4. Single-photon photophysical properties of **DL1** and **DZ1**.

Comp.	Solvents	λ_{max}[a]	$\log\varepsilon_{max}$[b]	λ_{max}[c]	Φ[d]	t/ns[e]
	Benzene	281	3.08	467	0.36	2.41
	DCM	279	2.84	507	0.35	2.72
	THF	280	3.16	497	0.36	2.58
**DL1**	Ethyl acetate	279	3.07	492	0.32	2.61
	Ethanol	280	3.08	526	0.17	2.08
	DMF	281	2.61	529	0.22	2.69
	Benzene	491	1.97	525	0.06	3.21
	DCM	494	4.21	571	<1%	3.20
	THF	467	2.00	567	<1%	3.18
**DZ1**	Ethyl acetate	466	1.98	565	<1%	3.25
	Ethanol	501	5.80	578	<1%	3.08
	DMF	481	1.96	574	<1%	3.14

[a] Peak position of the longest absorption band in nm.

[b] Maximum molar extinction coefficient.

[c] Peak position of SPEF, exited at the maximum wavelength of absorption.

[d] Fluorescence quantum yield.

[e] Fluorescence lifetime(ns).

Table S5. Two-photon absorption data for **DZ1**

Comp.	λ[a] (nm)	β[b] (cm/GM)	σ[c] (GM)
**DZ1**(DMSO,1mmol)	700	0.00879	414.36
**DZ1**(acetonitrile,0.1mmol)	700	0.00582	141.64
**DZ1+70 %**(0.1mmol)	700	0.0138	650.53
**DZ1+RNA**(1mmol)	700	0.0109	513.83

[a] Maximum nonlinear absorption wavelength.

[b] Two-photon absorption coeffcient.

[c] Two-photon absorption cross-section

Table S6. Three-photon absorption data for DZ1

Comp.	λ[a] (nm)	β[b] (cm^3/W^2)	σ[c] ($cm^6s^2photon^{-2}$)
**DZ1**(DMSO,1mmol)	1450	7.563×10^{-22}	2.073×10^{-74}
**DZ1**(acetonitrile,0.1mmol)	1450	5.506×10^{-22}	1.719×10^{-74}
**DZ1+70 %**(0.1mmol)	1450	1.021×10^{-21}	3.183×10^{-74}
**DZ1+RNA**(1mmol)	1450	9.954×10^{-22}	2.727×10^{-74}

[a] Maximum nonlinear absorption wavelength.

[b] Three-photon absorption coeffcient.

[c] Three-photon absorption cross-section

References

[1] S. V. Eliseeva, G. Aubock, F. V. Mourik, A. Cannizzo, B. Song, E. Deiters, A. S. Chauvin, M. Chergui and J. G. Bunzli, *J. Phys. Chem. B.,* 2010, **114**, 2932-2937.

[2] D. S. Corre, L. D. Boni, L. Misoguti, I. Cohanoschi, F. E. Hernandez and C. R. Mendonc, *Opt. Commun*., 2007, **277**, 440–445.
